# A combination of calcium hydroxide and sodium hydrosulphate controls pathogens causing environmental mastitis in recycled manure solids

**DOI:** 10.1186/s40643-024-00812-1

**Published:** 2024-10-08

**Authors:** Selladurai Praveen, Mukund A. Kataktalware, Priyanka Meena, Maharajan Lavanya, Priyanka Patoliya, Sakthivel Jeyakumar, Menon Rekha Ravindra, Mamta Chauhan, K. P. Ramesha, G. Letha Devi, John P. Kastelic, Arindam Dhali

**Affiliations:** 1 Livestock Production Management, Dairy Production Section, Southern Regional Station, ICAR- National Dairy Research Institute, Adugodi, Bengaluru, Karnataka 560030 India; 2 ICAR-National Institute of Animal Nutrition and Physiology, Adugodi, Bengaluru, Karnataka 560030 India; 3 Faculty of Veterinary Medicine, University of Calgary, AB, T2N 4Z6 Canada

**Keywords:** Conditioner combination, Depth, Properties, Manure bedding, Microbial biomass

## Abstract

Recycled manure solids (RMS) are dried cow dung processed using a manure dewatering machine and subsequently sun-dried to ~ 20% moisture. Benefits of RMS include abundant availability, low cost, and eco-friendliness, but its use as bedding material for cows is hindered by a moisture content that promotes microbial growth. This in vitro study evaluated impacts of calcium hydroxide (CH; 5 and 7.5%) and sodium hydrosulphate (SHS; 6 and 8%), independently and in combinations, at various depths of RMS, on physicochemical and microbial properties. The CH-treated groups had increased pH and reduced moisture on Day 0. Incorporating 7.5% CH + 6% SHS at 15–20 cm, and 7.5% CH + 8% SHS at all depths, effectively suppressed *Escherichia coli* and *Klebsiella spp.* Furthermore, a combination of 7.5% CH + 8% SHS at 20 cm inhibited coliform growth, whereas 7.5% CH with 6% SHS inhibited *Streptococcus spp*. In conclusion, a combination of 7.5% CH with either 6 or 8% SHS at a depth of 15 cm in RMS was particularly effective in controlling environmental mastitis-causing pathogens, specifically *E. coli* and *Klebsiella spp.*

**Graphical Abstract:**

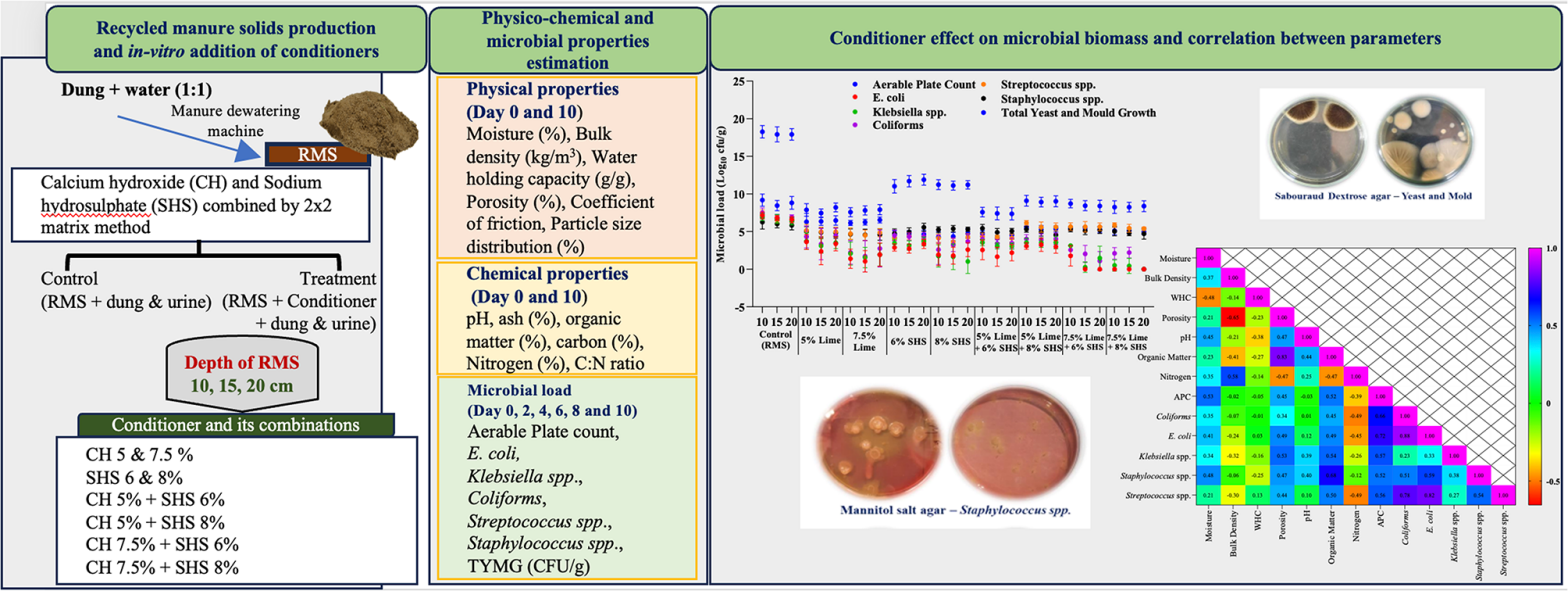

**Supplementary Information:**

The online version contains supplementary material available at 10.1186/s40643-024-00812-1.

## Introduction

Shelter and comfortable resting areas can alleviate physical and thermal discomfort for farm animals. Bedding can enhance physical comfort (Tuyttens 2005) plus general comfort; furthermore, it encourages resting, promotes production efficiency by improving udder health, milk quantity, and quality, and reduces injuries and lameness in dairy cows (Singh et al. 2020). Identifying cost-effective and sustainable materials to address increasing demands for bedding to promote animal welfare has made recycled manure solids (RMS) a potential alternative to conventional bedding materials.

Adoption of RMS, (i.e., “green bedding”) was pioneered in the United States in the 1970s (Timms 2008), based on using dairy waste solids and separated manure solids. More recently, products of anaerobic digestion of manure have also been used as bedding but there are greater production costs (Timms 2008; Gautam et al. 2020). Technical changes produced drier materials with 30% dry matter (adopted in European countries) (Leach et al. 2015).

Regarding RMS processing systems green manure, there are four major types: (1) pressed and dewatered; (2) digested and then pressed; (3) composted manure put into a rotating drum and exposing solids to air > 65 ^0^C for 1 d; and (4) mechanically dried for 12–15 min, at 370 °C at entry and 55 °C at exit, producing drier material with a lower clinical mastitis rate (Patel et al. 2019). Using dung for biogas production has promoted RMS as bedding. Advantages of RMS include availability, affordability, and environmental sustainability (Oultram and Bromley 2019), plus reductions in the cost of disposing manure. More recently, an important impetus to use RMS has been increased animal comfort (Zigo et al. 2020).

A limitation of RMS as bedding material is its moisture reduces water holding capacity and coefficient of friction, and increases bulk density, promoting microbial growth and reducing udder health, particularly if RMS contains environmental pathogens (e.g., *Klebsiella*,* and E. coli* (Ostrum et al. 2008). In one study (Black et al. 2014), compost bedded pack (CBP) in 42 barns contained high rates of *coliforms*, *E. coli*, *Streptococci*, *Staphylococci*, and *Bacillus spp.* Furthermore, RMS increased *Streptococcus thermophiles* and *Enterococcus* in raw milk (Rowbotham and Reugg 2016). Additional risks associated with RMS include increases in Johne’s disease and lameness (Ostrum et al. 2008).

Fresh RMS has a bacterial count ranging from 10^4^ to 10^8^ CFU/g (Green et al. 2014). Attempts to mitigate microbial growth include changes in pH, moisture reduction, and nutrient modifications, plus various conditioners and antimicrobial agents, e.g., calcium hydroxide, calcium silicate, sodium hydrosulphate and coal fly ash (Hogan et al. 1999; Anonymous 2008; Deskiharto et al. 2019; Rodrigues et al. 2021). Hydrated or slaked lime can be sparingly used on bedding materials to reduce microbial load but may damage teat or udder skin (Gautam et al. 2020). Alkaline conditioners, e.g., hydrated lime, inhibited growth of bacteria in animal bedding for 1 d (Hogan et al. 1999), whereas calcium silicate made bedding drier and reduced microbial growth (Deskiharto et al. 2019). Supplementation of broiler chick litter with sodium bisulfate (25 g/ft^2^) enhanced litter quality, improved growth rates of chicks, and significantly reduced ammonia emissions (Proch et al. 2021). Sodium hydrosulphate (SHS) a white, crystalline solid, strongly acidic (pH, 2.0-2.5) with antimicrobial activity due to reducing and bleaching properties (Anonymous 2008), was used as an acidic conditioner on kiln-dried solids and sawdust (Hogan et al. 1999, 2007), producing a pH of 4 (bacteriostatic). Furthermore, SHS is commonly used in poultry litter to bind ammonia and reduce bacteria (Choi and Moore 2008).

Despite numerous studies on impacts of chemical conditioners on microbial load in RMS, there is limited research on how these conditioners affect physical and chemical properties of RMS. Furthermore, effects of conditioner combinations at various depths within RMS on physicochemical properties and bacterial biomass have not been explored. This knowledge gap underscores the need for a comprehensive evaluation of how CH and SHS independently and in combination, influence physical, chemical, and microbial attributes of RMS bedding at various depths.

The current study is innovative, not only examining individual effects of CH and SHS, but also combined effects to assess potential synergistic benefits. By evaluating treatments at various depths and intervals, we aimed to provide a more nuanced understanding of how these conditioners optimized RMS bedding quality. The ultimate goal was to identify an optimal conditioning strategy that effectively controls microbial biomass, thereby improving the practicality and effectiveness of RMS for field applications.

## Materials and methods

### Study location

This study was conducted at the Animal Byproducts Utilization laboratory, Dairy Production Section, and Livestock Research Centre, Southern Regional Station of ICAR-National Dairy Research Institute, Adugodi, Bengaluru, India 12^0^57’ 01.0” N latitude and 77^0^ 36’ 17.8” E longitudes, 900 m above sea level. Minimum (winter) and maximum (summer) ambient temperatures are 13° and 34°C, respectively and average annual rainfall is ~ 100 cm, mostly from July to October.

### Production of RMS

Fresh dung was collected from the cattle yard and mixed 1:1 with water in a premixing tank; the resulting slurry was transferred to a manure-dewatering machine, a screw-press type equipped with a 3-phase induction motor and a 7.5 horsepower rating. The output capacity was 30–35 kg/h, with a final moisture content of 49–53%. The concentrated mass obtained from the dewatering machine was sundried for 20–24 h to attain 20–25% moisture, then packed and used for further study. Solids obtained from this process designated RMS or green RMS (Husfeldt et al. 2012).

### Design of in-vitro experiment

Calcium hydroxide (5 and 7.5%) and SHS (6 and 8%), independently and in combinations (CH 5% with 6% SHS, CH 5% with 8% SHS, CH 7.5% with 6% SHS, and CH 7.5% with 8% SHS) were mixed with RMS (Treatment) at various depths (10, 15, and 20 cm) and intentionally spiked with dung (24 g) and urine (10 ml) to mimic soiling of bedding material. Percentages of CH and SHS were based on our preliminary in vitro study with individual conditioners CH (5, 10, 15%), SHS (2, 5 and 8%), neem oil (5, 10, and 15%), coal fly ash (5, 10, and 15%), calcium silicate (5, 10, and 15%) and potassium hydroxide (1, 2 and 5%). As CH and SHS each reduced microbial load of RMS, for a combination study, lower concentrations were used, with expectations of synergism. As a control, RMS samples without conditioner were similarly treated. In each treatment group, dung and urine-soiled RMS groups were carefully removed daily and replaced with an equal amount of conditioner-mixed RMS every second day.

All groups were analyzed for physicochemical properties on Days 0 and 10, whereas bacteriological and fungal counts were enumerated on Days 0, 2, 4, 6, 8, and 10. Using individual conditioners, an antimicrobial effect was noticed up to 7 d, whereas Hogan et al. (2007) also used individual conditioners for 7 d. As the current study used combinations of conditioners to explore potential synergistic effects, a 10-d experimental duration was used. This reflects real-world scenarios, where farmers often replace RMS to manage excessive moisture while maintaining the desired bedding depth and it enabled assessment of cumulative impacts, addressing practical challenges in managing bedding.

### Sample collection and physicochemical analysis of RMS

Representative samples of the RMS were collected on Days 0 and 10 of treatment, and their physical and chemical properties were determined in triplicate. Moisture and ash were analyzed using standard protocols (AOAC 2012). The pH was determined using a pH probe inserted in a slurry prepared by mixing 5 g of sample in 50 mL distilled water (Husfeldt et al. 2012). The coefficient of friction was estimated by the fixed-funnel method (Al-Hashemi and Al-Amoudi 2018). Size distribution of RMS samples was determined with a vertical stack of sieves with aperture sizes of 4.75, 2.36, 1.18, 0.6, 0.3, and 0.15 mm (Jobim et al. 2007) and fineness modulus of RMS was calculated as described (Tapkire and Kumar 2023). Based on ash, organic matter and carbon were determined (Fournel et al. 2019). Water holding capacity, C:N ratio (total carbon divided by nitrogen), and porosity were estimated as described (Nayak and Kale 2020; Ferraz et al. 2020; and Khater 2015), respectively.

### Estimation of microbial load of RMS

An aliquot (10 g) of collected RMS samples (in duplicate) was mixed with 90 mL of sterilized PBS to create a 10¹ dilution. A 1 mL portion of this mixture was then transferred to 9 mL of diluent for further preparation. Selected dilutions were used to estimate various microbial loads. Aseptic precautions were taken during collection and processing of RMS samples. Nutrient agar, eosin methylene blue agar, McConkey agar, mannitol salt agar, and Edward medium (Himedia^®^) were used to enumerate arable plate counts of *E. coli* and *Klebsiella spp*., *Coliform spp*., *Staphylococcus spp*., and *Streptococcus spp*., respectively, with Sabouraud dextrose agar (Himedia^®^) used for total yeast and mold growth.

From a 10-fold dilution, 1 mL of inoculum was transferred to a Petri dish and for each inoculated plate, ~ 15–20 mL of sterile molten agar (Hi-media^®^) was poured and mixed with inoculum with gentle swirling movements. Petri plates were inverted and placed in incubators at 37 ºC for 48 h to assess growth of bacteria and 30 ºC for 3–5 d for yeast and mold. A colony counter was used to enumerate colonies. The number of organisms in a sample was calculated by multiplying mean colony count (duplicate plates) by the dilution factor and expressed as CFU/g. Mean colony count was calculated by arithmetic mean.

### Statistical analyses

All data were expressed as mean ± standard error of the mean (SEM). Assessment of in vitro stability in RMS at various intervals was done by repeated measures ANOVA using SPSS statistical software (Version 22.0; SPSS Inc., Chicago, IL, USA), with significance between days assessed using a multivariate ANOVA, with *post-hoc* comparisons done with Tukey’s multiple comparison test. The relationship between physical properties, chemical properties, and microbial load following the addition of conditioner combinations was assessed using a correlation matrix. A heat map was then generated using GraphPad Prism (Version 9.5.1, San Diego, CA, USA) to visually represent these correlations. For all analyses, *p* < 0.05 was considered significant. Figures were made using GraphPad Prism software.

## Results

### Effects of conditioners and combinations on physical properties of RMS

**Fig. 1:**
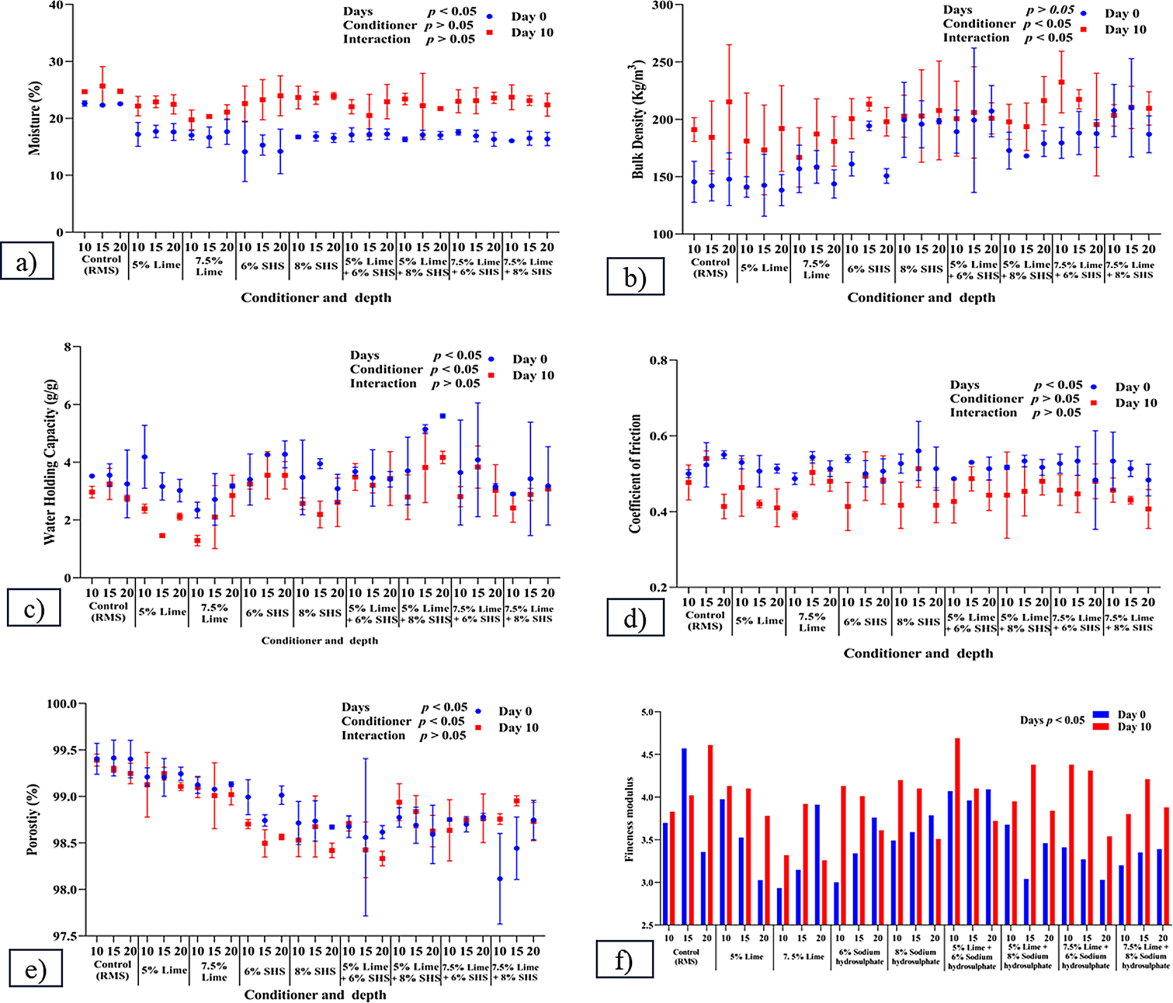
Physical properties of recycled manure solids after addition of conditioners and their combinations: (**a**) Moisture (%); (**b**) Bulk density (kg/m^3^); (**c**) Water holding capacity (g/g); (**d**) Coefficient of friction; (**e**) Porosity (%); and (**f**) Fineness modulus. All parameters were estimated with duplicate samples on Days 0 and 10. SHS = Sodium hydrosulphate

Impacts of conditioners on physical properties of RMS are summarized in Fig. 1a and f. Moisture of the Control at depths of 10 and 20 cm was higher (*P* < 0.03) on Day 10 (24.67 ± 0.15 and 24.75 ± 0.28, respectively) compared to Day 0 (22.61 ± 0.33 and 22.55 ± 0.12), (see Supplementary Material). Adding 6% SHS decreased (*p* = 0.0294) moisture at all depths compared to the Control on Day 0. There were increases in moisture on Day 10 in 5% CH at 15 cm (*p* = 0.04) and for 8% SHS at 10 (*p* = 0.04), 15 (*p* = 0.01), and 20 cm (*p* = 0.01). There were also increases in moisture in the following combinations: 5% CH + 8% SHS at 10 and 20 cm (*p* = 0.01); 7.5% CH + 6% SHS at 20 cm (*p* = 0.04); and 7.5% CH + 8% SHS at 10 (*p* = 0.03) and 15 cm (*p* = 0.02) depths of bedding.

Water-holding capacity (WHC) was not different (*p* = 0.8322) among Control, individual conditioner, and combinations of conditioner added to RMS on Day 0. Compared to Day 10, on Day 0, there was a decrease of WHC with: 5% CH at 15 cm (*p* = 0.03); 7.5% CH at 10 cm (*p* = 0.04); or 5% CH + 8% SHS at 20 cm (*p* = 0.01). On day 10, WHC increased with 7.5% CH + 6% SHS at 15 cm compared to 5% CH + 8% SHS at 10–15 cm (*p* = 0.03).

For bulk density (BD), there were no differences among conditioner groups and Control on Days 0 (*p* = 0.49) or 10 (*p* = 0.51).

There were no significant changes in the coefficient of friction of RMS (Control) at all depths on Day 10 compared to Day 0. However, there were significant decreases in coefficient of friction on Day 10 in control and 5% CH at 15 and 20 cm (*p* = 0.02), 7.5% CH + 6% SHS at 10 cm (*p* = 0.04), and 7.5% CH + 8% SHS at 15 cm (*p* = 0.003) of RMS compared to Day 0. There was no significant difference between the conditioner addition and Control group on Day 0. However, on Day 10, there was an increased coefficient of friction on Control RMS (0.54 ± 0.01) and 6% SHS at 15 cm compared to 5% CH + 8% SHS at 10 cm (see Supplementary Material). Overall, the coefficient of friction was lower on Day 10 versus 0.

There was a decrease in porosity on Day 10 compared to Day 0 with addition of 6 or 8% SHS at 20 cm (*p* = 0.021 and *p* = 0.043, respectively). On Day 0, there was a lower (*p* = 0.039) porosity with 7.5% CH + 8% SHS at 10 cm compared to control RMS. Furthermore, on Day 10, there was a significant decrease in porosity with 5% CH + 6% SHS, compared to control, at 15 and 20 cm, 5% CH at all depths, and 7.5% CH at 10 cm.

Conditioner combinations significantly reduced fineness modulus compared to the Control on Day 0. The overall fineness modulus among all groups was 3 to 4, with increased values on Day 10 compared to Day 0.

### Effect of addition of conditioners on chemical properties of RMS

**Fig. 2:**
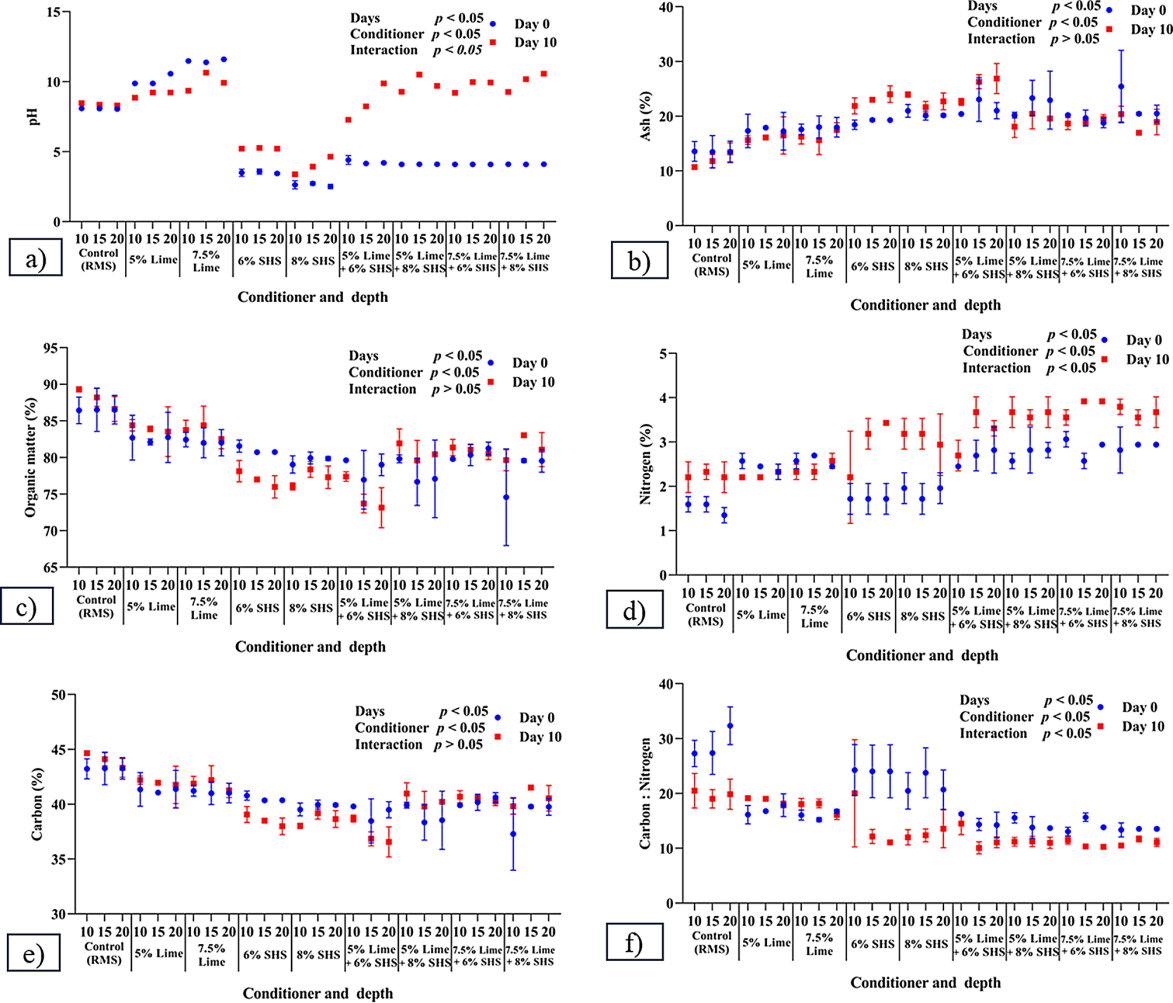
Chemical properties of recycled manure solids after addition of conditioners and their combinations: (**a**) pH; (**b**) Ash (%); (**c**) Organic matter (%); (**d**) Nitrogen (%); (**e**) Carbon (%); and (**f**) Carbon: Nitrogen ratio. All parameters were estimated with duplicate samples on Days 0 and 10. SHS = Sodium Hydrosulphate

Impacts of conditioners on chemical properties of RMS are in Fig. 2a and f. There was an increase (*p* = 0.001) in pH of RMS (Control) on Day 10 versus Day 0. Adding 8% SHS decreased pH compared to all other conditioners and their combinations and the Control group on both Days 0 and 10 (*p* = 0.001). Furthermore, 7.5% CH increased pH at all depths on Day 0 (*p* = 0.001). Adding SHS or a combination of CH + SHS decreased pH of RMS compared to control on Day 0 and vice versa on Day 10. A combination of CH + SHS significantly increased pH on Day 10 compared to Day 0.

There was less ash on Day 0 in control RMS compared to 7.5% CH + 8% SHS at 10 cm (*p* = 0.001). Adding 6 or 8% SHS or 5% CH + 6% SHS significantly increased ash compared to all other CH and SHS and combinations and Control RMS at all depths on Day 10. Both 15 and 20 cm had decreased ash in 7.5% CH with 6 or 8% SHS compared to 10 cm.

A significant decrease in OM and carbon content was noted on Day 10 with addition of 6% SHS at 15–20 cm, or 5% CH + 6% SHS at 10 cm compared to Day 0. Furthermore, on Day 0, there were significantly lower OM and carbon with 5% CH + 8% SHS at 15 cm and 7.5% CH + 8% SHS at 10 cm compared to Control at all depths. On Day 10, decreased OM and carbon were observed with: 6 or 8% SHS or 5% CH + 6% SHS at all depths; 5% CH + 8% SHS at 15 and 20 cm; 7.5% CH + 6% SHS at 20 cm; and 7.5% CH + 8% SHS at 10 cm. Regarding depth, there was increased organic matter (%) in 7.5% CH with 6 or 8% SHS at 15 or 20 versus 10 cm.

There was no significant change in nitrogen (%) and C: N ratio of Control RMS between days or among depths. However, there was a significant increase in nitrogen content with addition of conditioner combinations compared to Control RMS at all depths and days. Furthermore, on Days 0 and 10 there was a reduction (*p* = 0.001) in the C: N ratio with conditioner combinations compared to control RMS.

### Effects of addition of conditioners on microbial properties of RMS

**Fig. 3:**
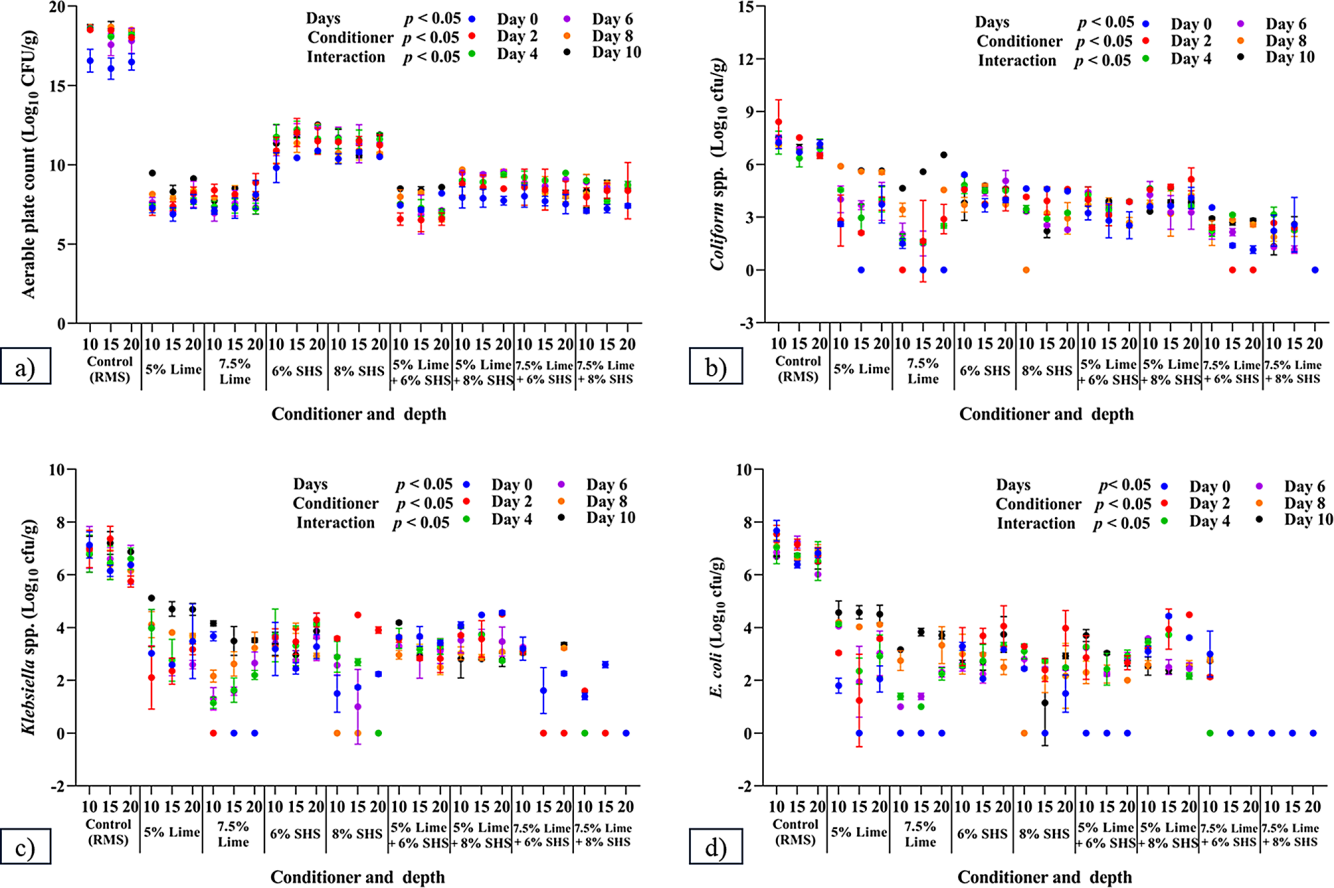
Microbial load in recycled manure solids on addition of conditioners and their combinations. (**a**) Aerable Plate count; (**b**) *Coliform* spp.; (**c**) *Klebsiella* spp.; and (**d**) *E. coli*. All parameters were estimated with duplicate samples on Days 0, 2, 4, 6, 8 and 10. SHS = Sodium Hydrosulphate

**Fig. 4:**
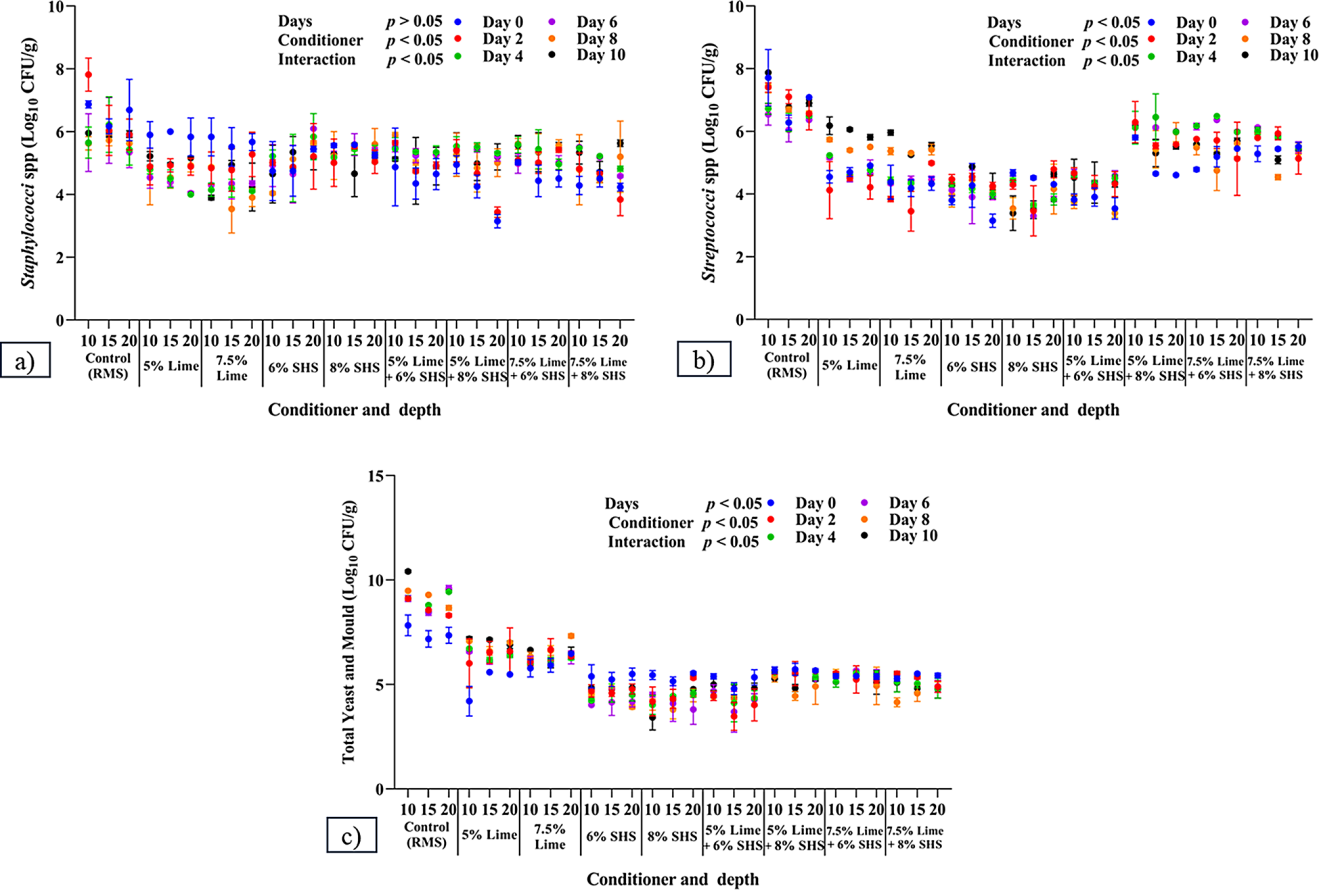
Microbial load in recycled manure solids after addition of conditioners and combinations: (**a**) *Staphylococcus* spp.; (**b**) *Streptococcus* spp.; and (**c**) Total Yeast and Mold growth. All parameters were estimated with duplicate samples on Days 0, 2, 4, 6, 8 and 10. SHS = Sodium Hydrosulphate

Impacts of conditioners and their combinations on the microbial load of RMS was assessed at a frequency of alternate days, with results depicted in Figs. 3 and 4.

#### Aerable plate count (APC)

On Day 0, CH at 5 or 7.5% and a combination of CH + SHS had lower (*P* < 0.001) Aerable Plate Counts (APC) compared to the Control and 6 or 8% SHS at all depths. Adding CH increased (*P* < 0.021) growth on Day 10 compared to other days at 10 cm (9.48 ± 0.00) and on Days 0, 4 and 6 at 15 cm. Adding 8% SHS at 20 cm resulted in more (*p* = 0.01) APC on Day 10 (11.89 ± 0.01) compared to Day 0 (10.50 ± 0.03).

Adding 5% CH + 8% SHS at 10 cm (7.94 ± 0.47) or 20 cm (7.73 ± 0.20); or 7.5% CH + 6% SHS at 20 cm; or 7.5% CH + 8% SHS at 15 cm, all reduced (*p* = 0.001) APC growth on Day 0 compared to all other days.

Adding 5% CH + 6% SHS significantly decreased APC on Day 0 at 15 cm compared to 10–20 cm, and on Day 8 at 20 cm compared to 10–15 cm. Adding 5% CH + 8% SHS significantly decreased APC on Day 4 at 10 and 15 cm compared to 20 cm. Adding 7.5% CH + 8% SHS significantly reduced APC on Day 4 at 15 cm versus 10–20 cm.

#### Total yeast and mold growth (TYMG)

The addition of CH, SHS, independently or in combinations, significantly reduced TYMG compared to control groups at all depths. However, no significant difference among addition of conditioner combinations was noted in the reduction of TYMG.

#### Coliform count

Conditioners and their combinations reduced (*p* = 0.001) the *coliform* count of RMS compared to control RMS on all days. Adding 7.5% CH + 8% SHS significantly reduced *coliform* growth at 20 cm on all days compared to the Control, 5% CH or 6% SHS, or 8% SHS at 15 and 20 cm; and 5% CH + 6% SHS, or 5% CH + 8% SHS (see Supplementary Material).

No *coliform* growth was observed with: 5% CH on Day 0 at 15 cm; 7.5% CH on Day 0 or 8 at 15 cm; on Day 0 and 6 at 20 cm; 8% SHS on Day 8 and 10 at 10 cm; 7.5% CH + 6% SHS on Day 2 at 15 cm; on Days 2, 4 and 6 at 20 cm; and 7.5% CH + 8% SHS on all days at 20 cm.

#### Escherichia coli

Conditioners or combinations reduced (*p* = 0.001) *E. coli* counts compared to Control RMS on all days. Significantly reduced or no *E. coli* growth was observed with: 5% CH at 15 cm on Day 0; 7.5% CH on Days 0 and 2 at 10, 15–20 cm; 8% SHS on Days 8 and 10 at 10 cm; 5% CH + 6% SHS at 10, 15–20 cm on Day 0; 7.5% CH + 6% SHS at 15–20 cm; and 7.5% CH + 8% SHS at 10, 15–20 cm on all days.

#### Klebsiella

The addition of conditioner and its combinations reduced (*p* = 0.001) *Klebsiella spp*. of RMS compared to Control RMS on all days. Significantly reduced or no *Klebsiella spp.* growth was observed with: 5% CH at 15 cm on Day 0; 7.5% CH on Days 0 and 2 at 15–20 cm; 8% SHS on Days 8 and 10 at 10–15 cm and Days 4 to 10 at 20 cm; 7.5% CH + 6% SHS at 15–20 cm on Days 2 to 10 and Days 2 to 6, respectively; and 7.5% CH + 8% SHS at 10, 15 on 20 cm on Days 1 to 4, Days 2 to 10, and on all days, respectively.

#### Streptococcus

Adding CH (5 or 7.5%), SHS (6 or 8%), and all combinations reduced (*p* = 0.001) growth of *Streptococcus spp.* on all days compared to control RMS at all depths. No significant difference among depths was observed for all conditioner combinations on all days. Adding 7.5% CH + 6% SHS to RMS reduced (*p* = 0.001) *Streptococcus* growth on: Day 0 at 10–15 cm; Day 8 at 15 cm; and 7.5% CH + 8% SHS on Day 0 and Day 8 compared to other days at 10–15 cm, respectively.

#### Staphylococcus

On Day 0, the conditioner combination reduced (*p* = 0.001) *Staphylococcus* count compared to Control at all depths. There was no significant difference among conditioner combinations on *Staphylococcus* growth on all days. Furthermore, no significant difference among depths was observed on control RMS, 7.5% CH, and 7.5% CH + 6% SHS on all days. Adding 7.5% CH + 8% SHS to RMS significantly decreased *Staphylococcus* growth on Days 4 and 6 at 20 cm.

### Correlation between physicochemical parameters and microbial biomass

**Fig. 5:**
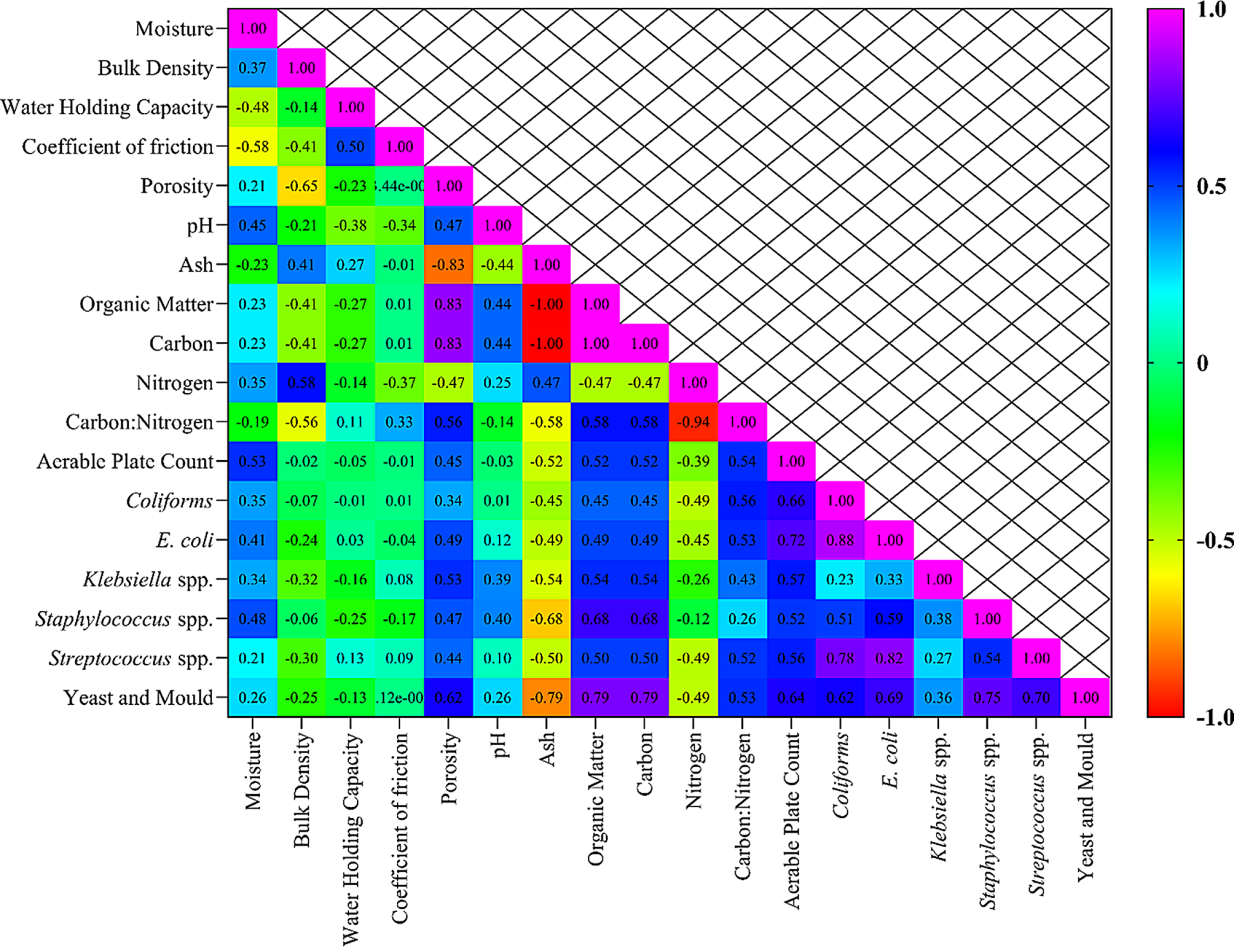
Heat map correlation matrix between various physical and chemical properties and microbial biomass

With increased RMS moisture, bulk density increased (*p* = 0.001) and there was decreased water holding capacity and coefficient of friction. Microbial biomass was positively correlated with moisture, bulk density, porosity, organic matter, pH, carbon, and C: N ratio, but ash percentage was negatively correlated with microbial load (Fig. 5).

## Discussion

Adding CH and/or SHS reduced moisture of RMS on Day 0, due to an exothermic reaction (Wong and Selvam 2009). Similarly, sodium bisulphate reduced moisture (%) of poultry litter (Johnson et al. 2021). Due to the hygroscopic nature, SHS absorbs moisture and tends to increase bedding moisture (Johnson et al. 2021), whereas melting of SHS crystals may increase moisture. However, as decreased bedding moisture was observed in the present study, further research is warranted to investigate impacts of SHS on bedding. On Day 10, all groups had a similar trend of moisture, although effects of conditioner may have been affected by dung and urine.

Water-holding capacity (WHC) depends on bedding moisture; lower moisture will hold more water, reducing bedding wetness. Water holding capacity of RMS was 1.04–1.18, 1.67–1.77 and 1.34–1.77 g/g in manure processed by a screw press, centrifuge, and roller press, respectively (Fournel et al. 2019). Reduced WHC was observed in conditioner-added RMS on Day 10 compared to Day 0. As WHC is inversely related to moisture content (Spiehs et al. 2013), on Day 10, there was increased moisture, so WHC was decreased. However, adding 7.5% CH + 6% SHS increased WHC on Day 10, as more CH absorbed moisture by an exothermic reaction, causing water loss (Wong and Selvam 2009).

Increased moisture will make particles aggregate, decreasing porosity (Ahn et al. 2008), whereas CH reduced moisture content of bedding (Hogan et al. 1999). In the current study, incorporation of CH and/or SHS significantly decreased moisture of RMS on Day 0.

Bulk density of RMS did not change significantly among groups. Bulk density had a positive correlation with moisture content, with increases in bulk density due to increased moisture and/or increased chemicals in combinations. On Day 10, an increase in the bulk density of the bedding was observed, with increased moisture (%).

The coefficient of friction (CoF) is the force required to move an object and inversely proportional to floor slipperiness (Sharma et al. 2019). The optimal coefficient of friction for cattle is 0.4–0.7 (Van der Tol et al. 2003). Cattle usually walk quickly on low-friction floors and more slowly on high-friction floors. This study had a coefficient of friction between 0.48 and 0.56 on Day 0 and 0.39 and 0.54 on Day 10; therefore, over time, the CoF of the bedding decreased, attributed to moisture from dung and urine.

With increased moisture (%) and bulk density, porosity will decrease (Cohen 2001). In this study, porosity values exceeded 95% across all groups, with moisture (%) ranging from 20 to 25%. Porosity was lower on Day 10 compared to Day 0, attributed to increased moisture and bulk density. Increased porosity on Day 0 with 6 or 8% SHS was attributed to reduced moisture (%). Reductions in porosity on Days 0 and 10 in conditioner combinations compared to control and CH were attributed to increased bulk density of bedding.

With conditioners and their combinations and an increase in the days, there was an increase in particles > 4 mm, perhaps due to particle aggregation from daily addition of dung and urine. There was a fine modulus of > 3.2 mm in all groups, which indicated that RMS is a mild-coarse particle. Coarse and medium particles absorb less water than fine particles (Spiehs et al. 2013). Furthermore, sand particles were considered mild coarse when fine modulus was 3.15 (Purwandito et al. 2017).

The pH of RMS has been reported as 8.5-9.0 (Fournel et al. 2019) and 9.16 ± 0.2 (Husfeldt et al. 2012). In the current study, CH increased the pH of RMS to alkaline, whereas SHS reduced pH to acidic, and their combination resulted in a slightly acidic pH on Days 0 and 10. In a previous report (Hogan et al. 1999), pH after addition of conditioners was 9.8 and 2.0-2.5 for CH and SHS, respectively. Increased pH with a combination versus SHS alone was attributed to the neutralizing effect of the combination.

As microbes need carbon to produce cell material and energy, a high content of carbon in a compost-bedded pack supports microbial growth (Kirchmann and Witter 1989). Furthermore, high carbon concentrations in organic components will immobilize nitrogen during decomposition (Ferraz et al. 2020). Adding CH reduced total organic carbon, due to the dilution effect of ash contents (Wong and Selvam 2009). Ash concentration depends on the presence of organic matter, which is negatively correlated (Larney et al. 2005); therefore, ash and organic matter act negatively together (Waqas et al. 2017). Since CH and SHS are inorganic, they increased ash and reduced organic matter and carbon content compared to Control on all days and depths. Total ash, OM, and carbon of RMS were 9.7, 90.4, and 45.1%, respectively in a static windrow, 10.5, 89.5, and 44.7% in a turned windrow, and 9.8, 90.0, and 45.1% in drum compost (Fournel et al. 2019).

Nitrogen is used by microorganisms for cell protein synthesis (Kirchmann 1985; Kirchmann and Witter 1989). Increasing moisture content and the C/N ratio will increase microbial load (Kupczyński et al. 2023). However, CH and/or SHS increased nitrogen content on all days and depths. This was attributed to an increase in nitrification with CH (Mkhonza et al. 2020), whereas with SHS, captured nitrogen increased nitrogen content (Choi and Moore 2008). Decreased carbon content of bedding due to inorganic conditioner and increased nitrogen content of bedding by CH and SHS reduced the C: N ratio.

The addition of CH and/or SHS significantly reduced total bacterial count (TBC), including *coliforms*, *E. coli*, *Klebsiella spp.*, *Staphylococcus spp.*, *Streptococcus spp.*, and total yeast and mold growth (TYMG) compared to the Control at 15 and 20 cm. Bacterial reductions after addition of CH were attributed to the high pH (Entry and Farmer 2001), reducing moisture (Wong and Selvam 2009), reducing the C: N ratio, increasing ash and nitrogen content, and reducing organic matter (Mushtaq et al. 2018). Furthermore, due to its bleaching and reducing properties, SHS reduced environmental loads of mastitis-causing pathogens (*E. coli* and *Klebsiella spp.*).

Results of adding CH and SHS to RMS were comparable to a report (Hogan and Smith 1997) that adding 1 kg CH to 10 kg of sawdust significantly reduced *Streptococci spp.* and gram-negative bacteria like *coliforms* and *Klebsiella spp*. Kiln-dried manure solids with ~ 1 kg of SHS reduced the growth of S*treptococcus spp.*, *Coliform*, *Klebsiella spp.* (Hogan et al. 2007). SHS reduced *Streptococci spp.* due to its antagonistic and bleaching properties (Anonymous 2008). Similarly, combinations like 7.5% CH + 6% SHS at 15–20 cm and 7.5% CH + 8% SHS at all depths had zero growth of *E. coli* and *Klebsiella spp.* on all days. This was attributed to an additive effect of CH and SHS that eliminated *E. coli* and *Klebsiella spp.*, as conditioners absorbed moisture from the thin peptidoglycan cell-walled bacteria (Silhavy et al. 2010), inactivating them. In contrast, efficacy against *Streptococcus* and *Staphylococcus spp*. was lower, potentially due to their thick-walled peptidoglycan.

Based on correlations, microbial biomass decreased in conditioner-added groups may have been due to reductions in moisture, porosity, organic matter and carbon, plus increases in ash and nitrogen. Reducing moisture produces a less porous material that hinders microbial adherence and proliferation (Cohen 2001).

Future research could explore the most effective and sustainable treatment duration for controlling microbial growth in RMS. Further, potential environmental consequences of using CH and SHS in RMS management must be assessed.

## Conclusion

Adding calcium hydroxide and/or sodium hydrosulphate decreased bedding moisture, and improved floor stability (coefficient of friction). The use of conditioners and their combinations increased ash content while reducing organic matter and carbon in recycled manure solids, which supported a reduction in microbial load. Specifically, adding 7.5% calcium hydroxide with 6% sodium hydrosulphate at 15 and 20 cm or 7.5% calcium hydroxide with 8% sodium hydrosulphate at all depths effectively suppressed growth of coliforms (i.e., *E. coli*, and *Klebsiella spp*). In conclusion, 7.5% calcium hydroxide combined with either 6 or 8% sodium hydrosulphate at 15 cm effectively controlled environmental mastitis-causing pathogens (*E. coli* and *Klebsiella spp.*) within recycled manure solids.

## Electronic supplementary material

Below is the link to the electronic supplementary material.

Supplementary Material 1

Supplementary Material 2

## Data Availability

Data will be made available upon reasonable request.
