# Supplementary material for: A combination of calcium hydroxide and sodium hydrosulphate controls pathogens causing environmental mastitis in recycled manure solids
Source: Bioresour Bioprocess. 2024 Oct 8;11(1):95. doi: 10.1186/s40643-024-00812-1 (PMC11461433; doi:10.1186/s40643-024-00812-1)
Supplement: Supplementary file 1 — Supplementary Material 1 [file 40643_2024_812_MOESM1_ESM.docx]

**Table 1. Effect of addition of individual conditioner on physical properties of RMS (Mean ± SEM)**

| **Conditioner** | **Depth** | **Moisture (%)** | | **WHC (g/g)** | | **Bulk density (Kg/m^3^)** | | **Coefficient of friction** | | **Porosity (%)** | |
| --- | --- | --- | --- | --- | --- | --- | --- | --- | --- | --- | --- |
|  |  | **0^th^ day** | **10^th^ day** | **0^th^ day** | **10^th^ day** | **0^th^ day** | **10^th^ day** | **0^th^ day** | **10^th^ day** | **0^th^ day** | **10^th^ day** |
| Control | 10 | 22.61±0.33^bA^ | 24.67±0.15^B^ | 3.52±0.02 | 2.96±0.14^ab^ | 145.53±10.26^A^ | 191.06±5.99^B^ | 0.5±0.01 | 0.47±0.03^abcde^ | 99.40±0.11^b^ | 99.39±0.04^e^ |
|  | 15 | 22.31±0.12^b^ | 25.67±2.41 | 3.55±0.28 | 3.25±0.38^ab^ | 142.01±7.54 | 184.31±18.30 | 0.52±0.03 | 0.54±0.01^e^ | 99.41±0.13^b^ | 99.29±0.02^de^ |
|  | 20 | 22.55±0.12^bA^ | 24.75±0.28^B^ | 3.25±0.83 | 2.75±0.08^ab^ | 147.86±13.24^A^ | 215.28±28.74^B^ | 0.55±0.0^A^ | 0.41±0.02^abcB^ | 99.40±0.14^b^ | 99.24±0.07^cde^ |
| 5% Lime | 10 | 17.18±1.48^ab^ | 22.15±1.21 | 4.18±0.77 | 2.39±0.10^ab^ | 141.14±5.15 | 181.08±24.17 | 0.53±0.01 | 0.46±0.04^abcd^ | 99.20±0.06^b^ | 99.12±0.24^bcde^ |
|  | 15 | 17.69±0.76^abA^ | 22.90±0.73^B^ | 3.16±0.33^A^ | 1.45±0.01^aB^ | 142.53±15.55^A^ | 173.37±22.55^B^ | 0.50±0.0^A^ | 0.42±0.01^abcdeB^ | 99.20±0.14^b^ | 99.24±0.04^cde^ |
|  | 20 | 17.60±1.05^ab^ | 22.46±1.19 | 3.02±0.27 | 2.11±0.08^ab^ | 138.31±7.82 | 191.98±21.54 | 0.51±0.0^A^ | 0.41±0.03^abB^ | 99.24±0.05^b^ | 99.11±0.03^bcde^ |
| 7.5% Lime | 10 | 17.03±0.55^ab^ | 19.74±1.21 | 2.34±0.19^A^ | 1.28±0.12^aB^ | 156.87±11.95 | 166.84±14.90 | 0.48±0.0^A^ | 0.39±0.01^abB^ | 99.12±0.06^b^ | 99.09±0.07^bcde^ |
|  | 15 | 16.66±1.27^ab^ | 20.33±0.22 | 2.71±0.63 | 2.09±0.77^ab^ | 158.60±8.20 | 187.30±17.72 | 0.54±0.01 | 0.50±0.02^abcde^ | 99.07±0.01^ab^ | 99.01±0.25^abcde^ |
|  | 20 | 17.64±1.56^ab^ | 21.09±0.92 | 3.17±0.05 | 2.84±0.50^ab^ | 143.73±7.13 | 180.74±12.52 | 0.51±0.01 | 0.48±0.02^bcde^ | 99.13±0.01^ab^ | 99.01±0.07^abcde^ |
| 6% Sodium Hydrosulphate | 10 | 14.14±3.69^a^ | 22.59±2.18 | 3.40±0.63 | 3.24±0.12^ab^ | 161.05±6.06^A^ | 200.58±10.08^B^ | 0.54±0.0^A^ | 0.41±0.04^abcdeB^ | 98.99±0.13^ab^ | 98.70±0.03^abcde^ |
|  | 15 | 15.29±1.25^a^ | 23.27±2.49 | 4.26±0.01 | 3.55±0.57^ab^ | 194.37±2.34^A^ | 213.21±3.39^B^ | 0.50±0.01 | 0.49±0.04^e^ | 98.74±0.04^ab^ | 98.49±0.10^ab^ |
|  | 20 | 14.20±2.76^a^ | 23.95±2.48 | 4.27±0.33 | 3.54±0.33^ab^ | 150.76±3.67^A^ | 197.93±7.15^B^ | 0.50±0.01 | 0.48±0.04^de^ | 99.01±0.06^abA^ | 98.56±0.02^abcdB^ |
| 8% sodium Hydrosulphate | 10 | 16.72±0.22^abA^ | 23.65±1.41^B^ | 3.47±0.91 | 2.56±0.14^ab^ | 199.56±18.87 | 202.74±10.51 | 0.52±0.01 | 0.41±0.04^abcde^ | 98.71±0.16^ab^ | 98.52±0.12^abc^ |
|  | 15 | 16.81±0.56^abA^ | 23.56±0.78^B^ | 3.94±0.12^A^ | 2.18±0.32^abB^ | 195.78±11.80 | 202.91±23.20 | 0.56±0.04 | 0.51±0.03^abcde^ | 98.73±0.15^ab^ | 98.67±0.23^abcde^ |
|  | 20 | 16.55±0.55^abA^ | 23.92±0.42^B^ | 3.08±0.35 | 2.61±0.59^ab^ | 198.37±1.327 | 207.75±24.85 | 0.51±0.03 | 0.41±0.03^abc^ | 98.67±0.01^abA^ | 98.42±0.05^abB^ |
| 5% Lime + 6% Sodium Hydrosulphate | 10 | 17.11±0.86^ab^ | 22.04±0.89 | 3.68±0.10 | 3.49±0.32^ab^ | 189.24±10.79 | 200.54±18.86 | 0.48±0.01 | 0.42±0.03^abc^ | 98.67±0.08^ab^ | 98.71±0.05^abcde^ |
|  | 15 | 17.19±0.69^ab^ | 20.50±2.62 | 3.45±0.69 | 3.20±0.18^ab^ | 199.27±36.30 | 206.01±22.94 | 0.53±0.00 | 0.48±0.02^abcde^ | 98.56±0.59^ab^ | 98.42±0.21^ab^ |
|  | 20 | 17.23±0.62^ab^ | 22.92±2.13 | 3.40±0.19 | 3.43±0.66^ab^ | 207.15±12.88 | 200.86±7.84 | 0.51±0.02 | 0.44±0.02^abcd^ | 98.61±0.04^ab^ | 98.33±0.06^a^ |
| 5% Lime + 8% Sodium Hydrosulphate | 10 | 16.31±0.29^abA^ | 23.4±0.71^B^ | 3.69±0.82 | 2.79±0.54^ab^ | 172.81±9.26 | 197.83±8.78 | 0.51±0.01 | 0.44±0.06^a^ | 98.77±0.07^ab^ | 98.93±0.14^abcde^ |
|  | 15 | 17.09±0.58^ab^ | 22.23±4.01 | 5.14±0.10 | 3.82±0.86^b^ | 168.09±1.01 | 193.58±11.94 | 0.53±0.01 | 0.45±0.03^abcd^ | 98.69±0.13^ab^ | 98.83±0.12^abcde^ |
|  | 20 | 17.01±0.46^abA^ | 21.72±0.18^B^ | 5.60±0.05^A^ | 4.16±0.15^bB^ | 178.78±6.41 | 216.29±12.13 | 0.51±0.01 | 0.48±0.02^abcde^ | 98.59±0.22^ab^ | 98.62±0.11^abcd^ |
| 7.5 % Lime + 6% Sodium Hydrosulphate | 10 | 17.54±0.34^ab^ | 23.00±1.43 | 3.64±1.28 | 2.80±0.24^ab^ | 179.44±7.75 | 232.49±15.48 | 0.52±0.01 | 0.45±0.02^abcde^ | 98.75±0.01^ab^ | 98.63±0.23^abcd^ |
|  | 15 | 16.91±0.71^ab^ | 23.10±1.61 | 4.08±1.39 | 3.83±0.51^b^ | 188.08±10.89^A^ | 217.42±4.86^B^ | 0.53±0.02 | 0.44±0.02^abcde^ | 98.70±0.05^ab^ | 98.74±0.01^abcde^ |
|  | 20 | 16.33±0.86^abA^ | 23.59±0.69^B^ | 3.15±0.07 | 3.02±0.62^ab^ | 187.63±6.94 | 195.44±25.86 | 0.48±0.07 | 0.48±0.03^cde^ | 98.78±0.02^ab^ | 98.76±0.18^abcde^ |
| 7.5% Lime + 8% Sodium Hydrosulphate | 10 | 16.05±0.08^abA^ | 23.71±1.54^B^ | 2.90±0.04 | 2.41±0.35^ab^ | 207.62±13.13 | 203.48±5.42 | 0.53±0.44 | 0.45±0.02^abcde^ | 98.11±0.34^a^ | 98.75±0.043^abcde^ |
|  | 15 | 16.50±0.86^abA^ | 23.11±0.59^B^ | 3.42±1.38 | 2.88±0.14^ab^ | 210.09±24.6^A^ | 210.44±10.64^B^ | 0.51±0.0^A^ | 0.43±0.01^abcdeB^ | 98.44±0.23^ab^ | 98.95±0.037^abcde^ |
|  | 20 | 16.37±0.83^ab^ | 22.37±1.41 | 3.18±0.95 | 3.07±0.01^ab^ | 187.00±9.27 | 209.59±8.39 | 0.48±0.02 | 0.41±0.02^abcde^ | 98.74±0.14^ab^ | 98.73±0.14^abcde^ |

Means with different superscripts within rows (capital letters) and columns (small letters) differ significantly (*p* < 0.05) between days and conditioner, respectively.

**Table 2. Effect of addition of individual conditioner on chemical properties of RMS (Mean ± SEM)**

| **Conditioner** | **Depth** | **pH** | | **Ash (%)** | | **Organic matter (%)** | | **Carbon (%)** | |
| --- | --- | --- | --- | --- | --- | --- | --- | --- | --- |
|  |  | **0^th^ day** | **10^th^ day** | **0^th^ day** | **10^th^ day** | **0^th^ day** | **10^th^ day** | **0^th^ day** | **10^th^ day** |
| Control | 10 | 8.08±0.08^eA^ | 8.46±0.01^fB^ | 13.55±1.28^a^ | 10.69±0.24^a^ | 86.43±1.28^b^ | 89.30±0.24^h^ | 43.21±0.64^b^ | 44.65±0.12^h^ |
|  | 15 | 8.07±0.01^eA^ | 8.36±0.00 ^gB^ | 13.48±2.09^a^ | 11.79±0.86^ab^ | 86.51±2.09^b^ | 88.20±0.86^gh^ | 43.25±1.04^b^ | 44.10±0.43^gh^ |
|  | 20 | 8.03±0.02^eA^ | 8.30±0.01^gB^ | 13.47±1.38^a^ | 13.38±1.20^abc^ | 86.52±1.38^b^ | 86.61±1.20^fgh^ | 43.26±0.69^b^ | 43.30±0.61^fgh^ |
| 5% Lime | 10 | 9.87±0.01^fA^ | 8.85±0.01^hB^ | 17.31±2.16^ab^ | 15.60±0.54^abcd^ | 82.68±2.17^ab^ | 84.39±0.54ef^gh^ | 41.34±1.08^ab^ | 42.19±0.27^efgh^ |
|  | 15 | 9.87±0.01^fA^ | 9.21±0.01^iB^ | 17.89±0.30^abA^ | 16.10±0.27^abcdeB^ | 82.10±0.30^ab^ | 83.89±0.27^defgh^ | 41.05±0.15^abA^ | 41.94±0.14^defghB^ |
|  | 20 | 10.56±0.01^gA^ | 9.22±0.01^iB^ | 17.25±2.42^ab^ | 16.47±2.41^abcde^ | 82.74±2.42^ab^ | 83.52±2.40^defgh^ | 41.37±1.21^ab^ | 41.76±1.20^defgh^ |
| 7.5% Lime | 10 | 11.48±0.01^hA^ | 9.35±0.01^iB^ | 17.56±0.69^ab^ | 16.24±0.94^abcde^ | 82.43±0.69^ab^ | 83.75±0.94^defgh^ | 41.21±0.34^ab^ | 41.87±0.47^defgh^ |
|  | 15 | 11.38±0.01^hA^ | 10.64±0.01^mB^ | 18.01±1.43^ab^ | 15.59±1.84^abcd^ | 81.98±1.44^ab^ | 84.40±1.84^efgh^ | 40.99±0.71^ab^ | 42.20±0.92^efgh^ |
|  | 20 | 11.59±0.02^hA^ | 9.91±0.01^kB^ | 17.98±1.26^ab^ | 17.49±0.92^bcdef^ | 82.01±1.27^ab^ | 82.50±0.92^cdefg^ | 41.00±0.63^ab^ | 41.25±0.46^cdefg^ |
| 6% Sodium Hydrosulphate | 10 | 3.49±0.15^bA^ | 5.21±0.05^dB^ | 18.44±0.58^ab^ | 21.88±1.03^efgh^ | 81.55±0.58^ab^ | 78.11±1.03^abcd^ | 40.77±0.29^ab^ | 39.05±0.51^abcd^ |
|  | 15 | 3.57±0.12^bcA^ | 5.27±0.01^dB^ | 19.29±0.16^abA^ | 23.01±0.21^fghB^ | 80.70±0.16^abA^ | 76.99±0.21^abcB^ | 40.35±0.08^abA^ | 38.49±0.11^abcB^ |
|  | 20 | 3.44±0.04^bA^ | 5.21±0.05^dB^ | 19.26±0.02^abA^ | 24.02±1.07^ghB^ | 80.73±0.03^abA^ | 75.97±1.07^abB^ | 40.36±0.01^ab^ | 37.98±0.54^ab^ |
| 8% sodium Hydrosulphate | 10 | 2.62±0.16^aA^ | 3.38±0.02^aB^ | 20.96±0.83^ab^ | 23.96±0.34^gh^ | 79.03±0.83^ab^ | 76.03±0.34^ab^ | 39.51±0.41^ab^ | 38.01±0.17^ab^ |
|  | 15 | 2.72±0.06^aA^ | 3.93±0.06^bB^ | 20.08±0.57^ab^ | 21.65±0.76^defgh^ | 79.91±0.57^ab^ | 78.34±0.76^abcde^ | 39.95±0.28^ab^ | 39.17±0.38^abcde^ |
|  | 20 | 2.51±0.09^aA^ | 4.64±0.02^cB^ | 20.15±0.19^ab^ | 22.71±1.08^fgh^ | 79.84±0.20^ab^ | 77.29±1.08^abc^ | 39.92±0.09^ab^ | 38.64±0.54^abc^ |
| 5% Lime + 6% Sodium Hydrosulphate | 10 | 4.4±0.18^dA^ | 7.27±0.01^eB^ | 20.39±0.01^abA^ | 22.61±0.45^fghB^ | 79.60±0.02^abA^ | 77.38±0.45^abcB^ | 39.80±0.01^abA^ | 38.69±0.23^abcB^ |
|  | 15 | 4.15±0.03^dA^ | 8.23±0.01^gB^ | 23.05±2.83^ab^ | 26.30±0.90^h^ | 76.94±2.83^ab^ | 73.69±0.90^a^ | 38.47±1.41^ab^ | 36.84±0.45^a^ |
|  | 20 | 4.19±0.04^dA^ | 9.86±0.01^kB^ | 21.00±1.04^ab^ | 26.87±1.93^h^ | 78.99±1.04^ab^ | 73.12±1.93^a^ | 39.49±0.52^ab^ | 36.56±0.97^a^ |
| 5% Lime + 8% Sodium Hydrosulphate | 10 | 4.08±0.02^cdA^ | 9.28±0.03^iB^ | 20.18±0.38^ab^ | 18.07±1.39^cdefg^ | 79.81±0.38^ab^ | 81.92±1.39^bcdef^ | 39.90±0.19^ab^ | 40.96±0.69^bcdef^ |
|  | 15 | 4.09±0.01^cdA^ | 10.5±0.01^mB^ | 23.33±2.28^b^ | 20.42±0.95^defg^ | 76.66±2.28^a^ | 79.57±1.95^bcd^ | 38.33±1.14^a^ | 39.78±0.97^bcde^ |
|  | 20 | 4.09±0.01^cdA^ | 9.69±0.01^jB^ | 22.92±3.74^ab^ | 19.56±0.19^defg^ | 77.07±3.74^ab^ | 80.43±0.19^bcde^ | 38.53±1.87^ab^ | 40.21±0.09^bcde^ |
| 7.5 % Lime + 6% Sodium Hydrosulphate | 10 | 4.09±0.01^cdA^ | 9.2±0.02^dB^ | 20.21±0.13^ab^ | 18.64±0.78^cdefg^ | 79.78±0.13^ab^ | 81.35±0.78^bcdef^ | 39.89±0.06^ab^ | 40.67±0.39^bcdef^ |
|  | 15 | 4.09±0.01^cdA^ | 9.97±0.01^kB^ | 19.65±1.04^ab^ | 18.94±0.46^cdefg^ | 80.34±1.04^ab^ | 81.05±0.46^bcdef^ | 40.17±0.52^ab^ | 40.52±0.23^bcdef^ |
|  | 20 | 4.08±0.01^cdA^ | 9.93±0.01^kB^ | 18.76±0.61^ab^ | 19.50±0.56^defg^ | 81.23±0.61^ab^ | 80.49±0.55^bcde^ | 40.61±0.30^ab^ | 40.24±0.28^bcde^ |
| 7.5% Lime + 8% Sodium Hydrosulphate | 10 | 4.09±0.01^cdA^ | 9.26±0.02^hB^ | 25.43±4.67^b^ | 20.37±1.01^defg^ | 74.56±4.67^a^ | 79.62±1.01^bcde^ | 37.28±2.33^a^ | 39.81±0.51^bcde^ |
|  | 15 | 4.08±0.01^cdA^ | 10.17±0.01^lB^ | 20.44±0.19^abA^ | 16.97±0.17^bcdefB^ | 79.55±0.19^abA^ | 83.02±0.17^cdefgB^ | 39.77±0.09^abA^ | 41.51±0.08^cdefgB^ |
|  | 20 | 4.09±0.01^cdA^ | 10.56±0.01^mB^ | 20.48±1.08^ab^ | 18.92±1.64^cdefg^ | 79.51±1.08^ab^ | 81.07±1.64^bcdef^ | 39.75±0.54^ab^ | 40.53±0.82^bcdef^ |

Means with different superscripts within rows (capital letters) and columns (small letters) differ significantly (*p* < 0.05) between days and conditioner, respectively.


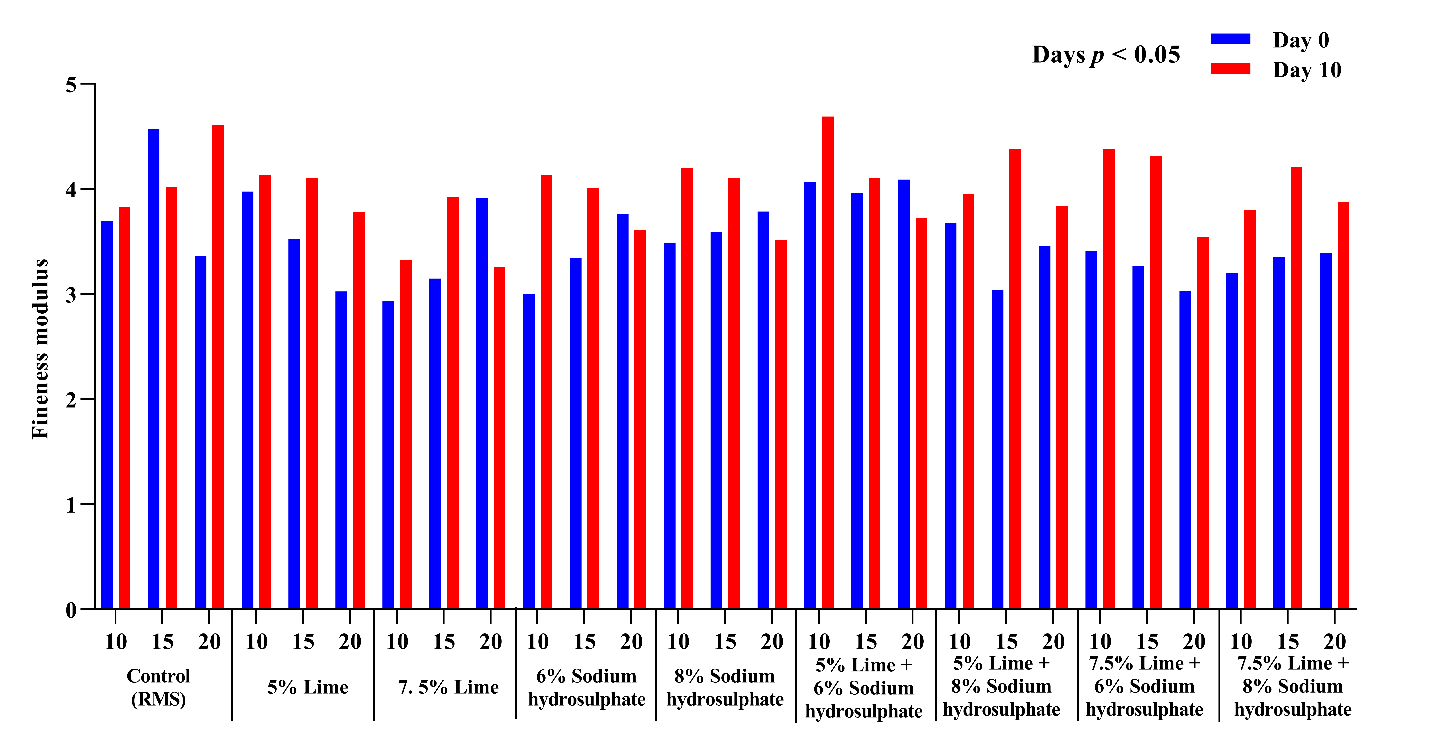


**Fig. 1 Fineness modulus of RMS (control) and conditioner combination added RMS on day 0 and 10**


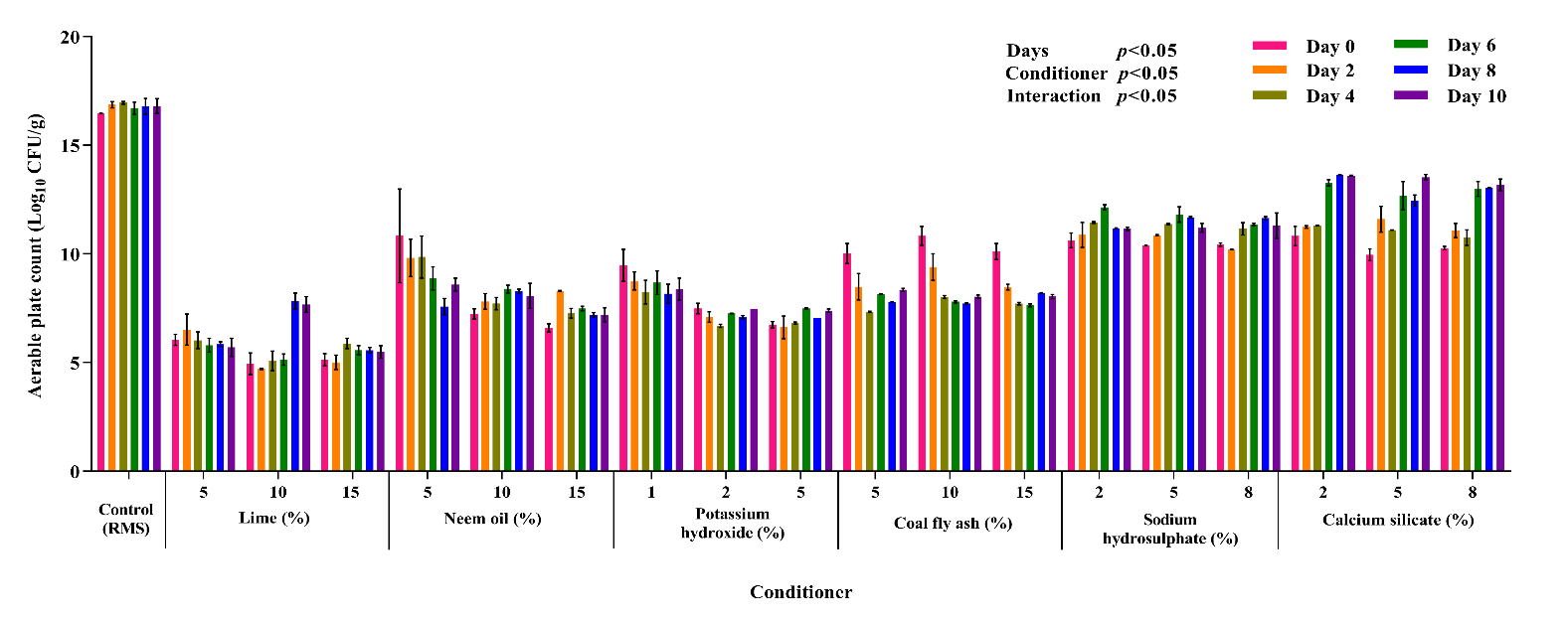

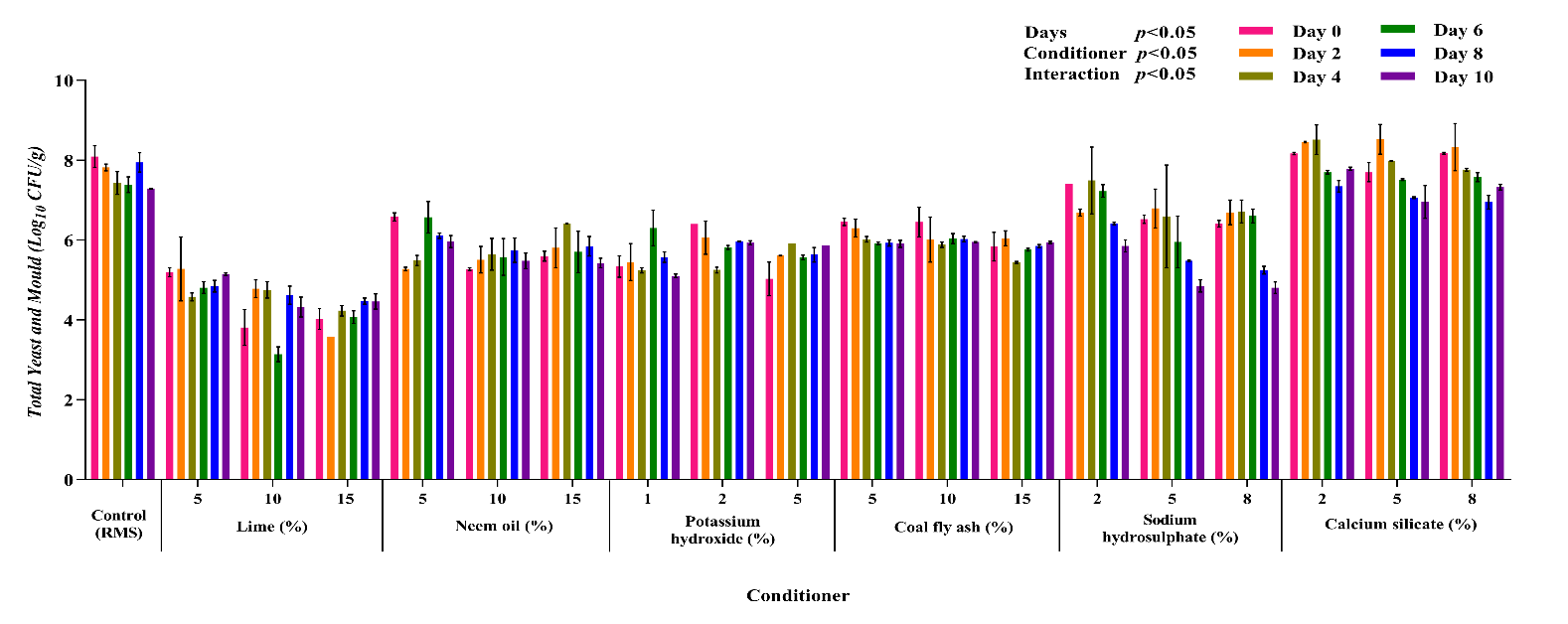


**a**

**b**

**Fig. 2**. **Effect of addition of different levels of conditioners in aerable plate count (a) and total yeast and mold growth (b)**


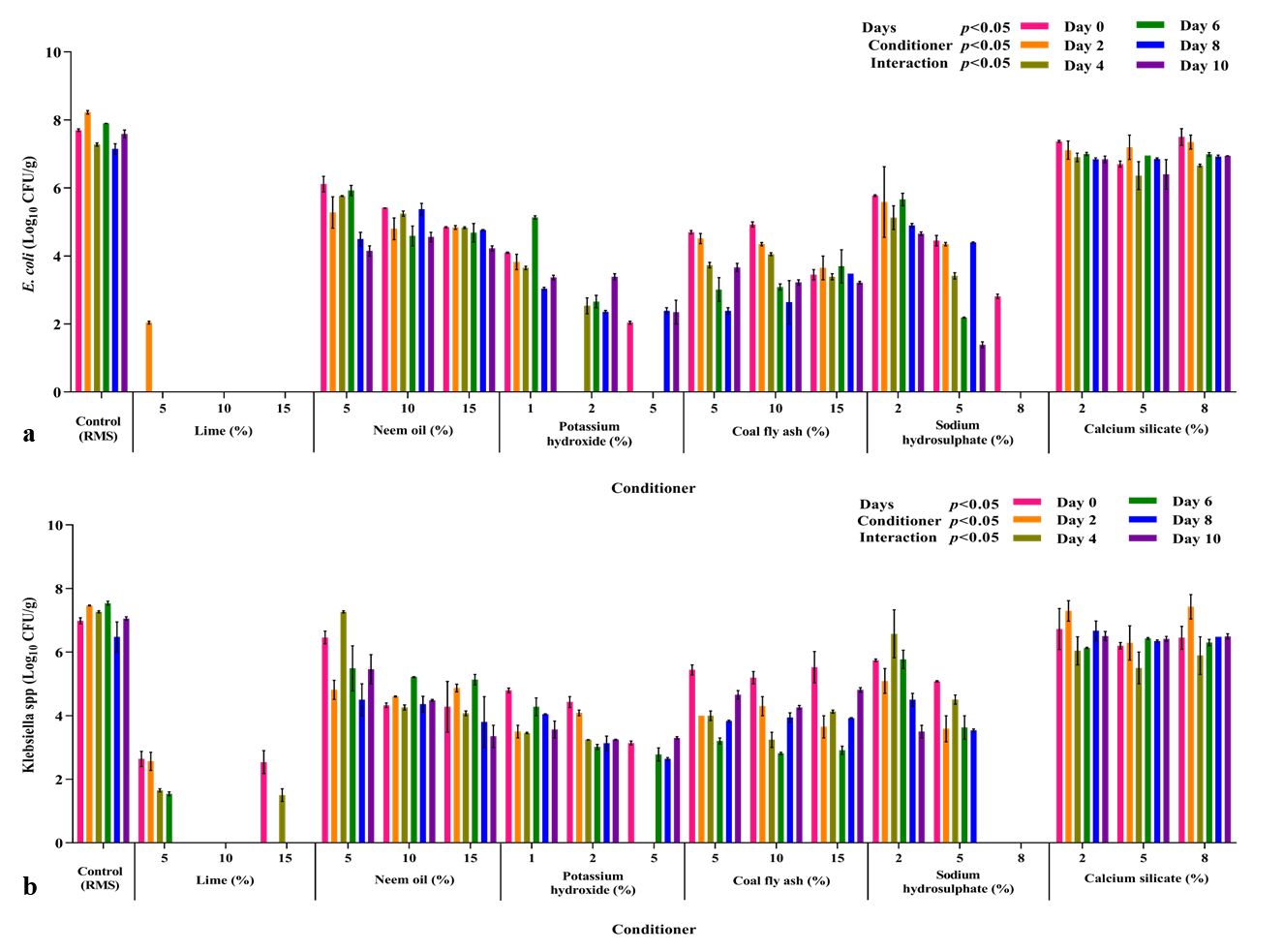


**Fig. 3. Effect of addition of different levels of conditioners in *E. coli* (a) and *Klebsiella* *spp.* count (b)**


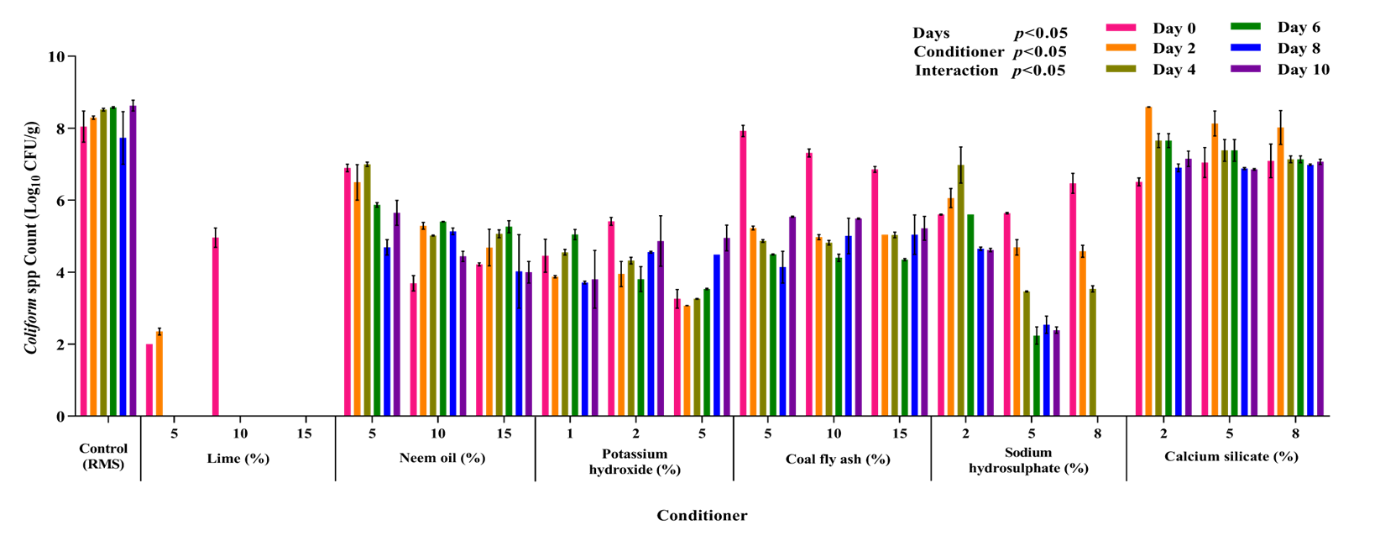


**Fig. 4**. **Effect of addition of different levels of conditioners in *Coliform spp.* count**


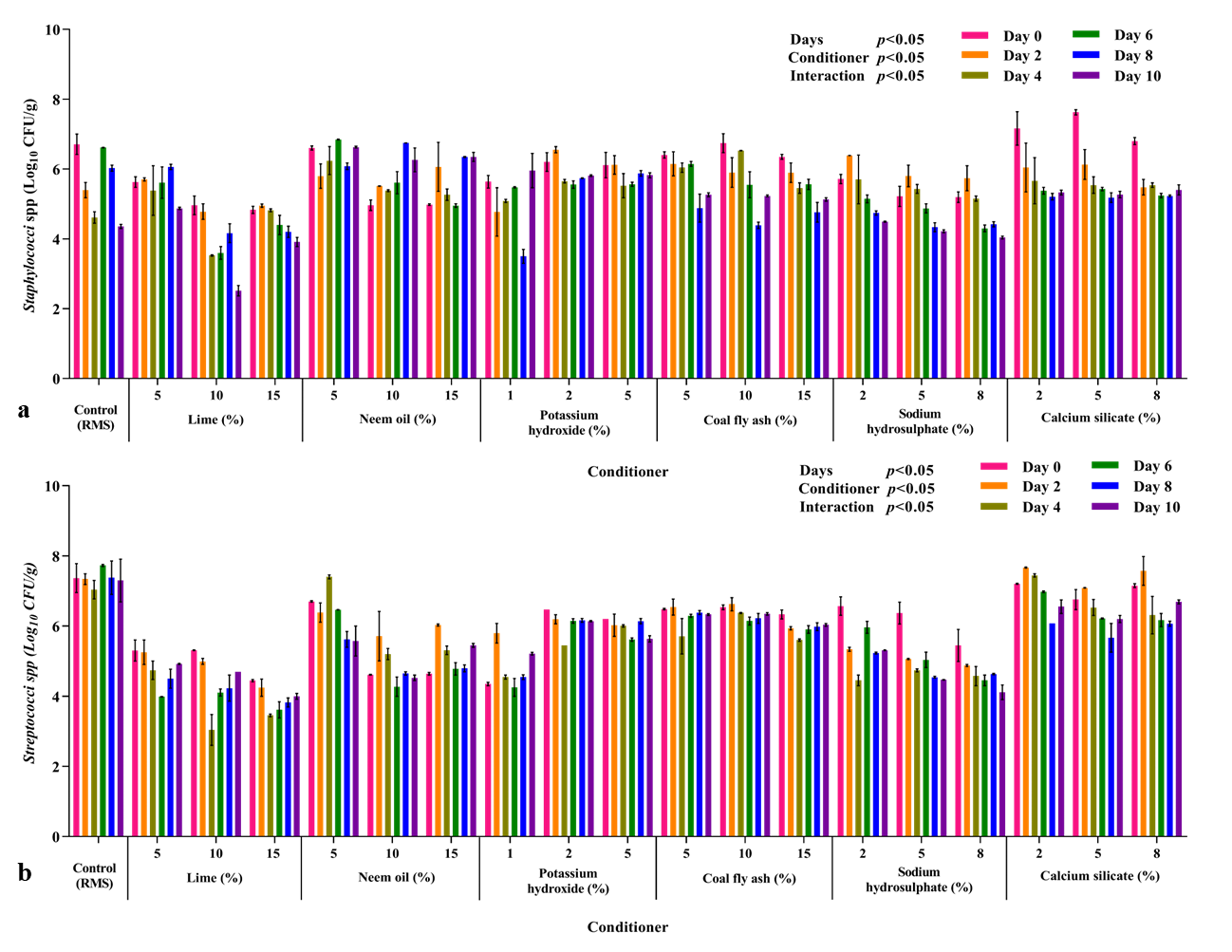


**Fig. 5**. Effect of addition of different levels of conditioners in *Streptococcus spp.* (a) and *Staphylococcus spp.* count (b).
