# Supplementary figures and images for: A combination of calcium hydroxide and sodium hydrosulphate controls pathogens causing environmental mastitis in recycled manure solids
Source: Bioresour Bioprocess. 2024 Oct 8;11(1):95. doi: 10.1186/s40643-024-00812-1 (PMC11461433; doi:10.1186/s40643-024-00812-1)

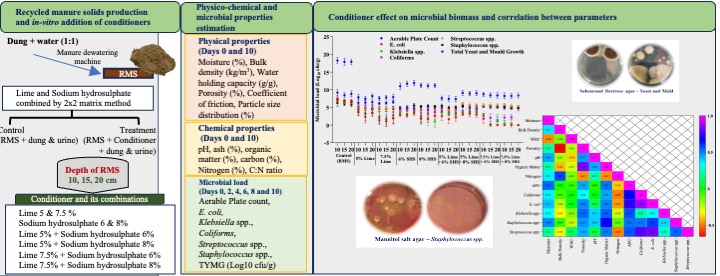

Supplement: Supplementary file 2 — Supplementary Material 2 [file 40643_2024_812_MOESM2_ESM.jpeg]
